# Cellular and molecular keys to entry: Mechanisms mediating *Orthoflavivirus* infection of the mosquito midgut

**DOI:** 10.1371/journal.ppat.1013617

**Published:** 2025-10-27

**Authors:** Godfrey Nattoh, Philip M. Armstrong, Doug E. Brackney

**Affiliations:** Department of Entomology, The Connecticut Agricultural Experiment Station, New Haven, Connecticut, United States of America; University of Iowa, UNITED STATES OF AMERICA

Mosquitoes are the primary vector for numerous *Orthoflaviviruses* (*Flaviviridae*), including dengue virus (DENV), Zika virus (ZIKV), West Nile virus (WNV), Japanese encephalitis virus (JEV), and yellow fever virus (YFV). These viruses pose a significant threat to global human health, resulting in widespread epidemics that cause considerable morbidity and mortality worldwide. Given the lack of effective treatments or vaccines for many of these pathogens, current disease control efforts predominantly focus on vector management using insecticides, which are inherently limited. Gaining an understanding of the early events mediating virus-vector interactions will be essential for developing novel control strategies targeting this stage of the transmission cycle.

Vector competence is the ability of an arthropod vector to become infected with a pathogen, permit replication, and ultimately transmit the pathogen to a new host. To be successfully transmitted to a new host, viruses must overcome multiple barriers to infection within the mosquito host; these barriers include the midgut infection barrier, midgut escape barrier, the salivary glands infection barrier, and the salivary glands escape barrier. Several factors such as vector immunity, cellular, and/or molecular virus-vector incompatibility, receptor availability, and structural obstacles are thought to contribute to these barriers; however, very little is known about the nature of these barriers.

The midgut is the first tissue encountered by arboviruses and successful transmission hinges on the virus’s ability to not only establish infection but also escape the midgut. The mosquito midgut is composed of roughly 5,000–10,000 cells [1–4]. During laboratory-based infection assays, mosquitoes will ingest 1,000s of virus particles; however, this estimate can vary depending on the viremic dose, volume of blood, mosquito species, viral species and strain, and virus-vector pairing. Previous studies with WNV and Venezuelan equine encephalitis virus (VEEV; *Togaviridae*, *Alphavirus*) have demonstrated that only a limited number of these cells (~20–80) become infected upon ingestion of an infectious blood meal [3,4]. Additionally, when using a mixed population of VEEV marked with green (GFP) and cherry fluorescent protein (CFP) at equal concentrations, it was demonstrated that about a third of all infected cells were expressing GFP-VEEV, another third with CFP-VEEV, and the last third infected with both particles [4]. Probabilistically, it is highly unlikely that a third of these relatively few infected cells would be dually infected if cells were infected entirely at random. This raises the possibility that only a handful of midgut epithelial cells are initially susceptible to infection.

Orthoflavivirus infection in the mosquito midgut is not completely stochastic, but the exact reasons for variations in infection outcomes remain unknown. This article explores our current understanding of how orthoflaviviruses interact with the midgut, focusing on the early stages of infection. We will examine the roles that cellular receptors, midgut cell types, the orthoflaviviruses nonstructural protein 1 (NS1), and *Wolbachia* have on shaping infection outcomes.

## Cellular receptors: Hunting for the culprit

**Fig 1:**
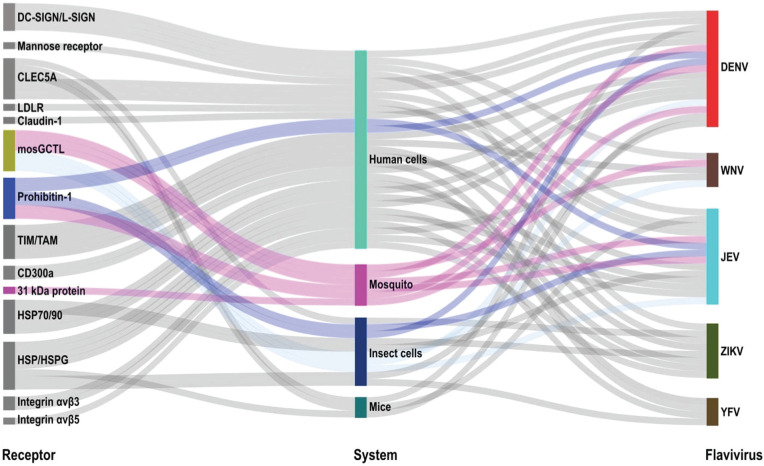
Orthoflaviviruses exploit multiple putative receptors to initiate infection. Sankey plot highlighting the diversity of putative receptors (left) associated with orthoflavivirus infection (right) and the experimental systems from whence they were identified across diverse cellular systems (center). The highlighted flows represent the putative receptors identified in mosquito vector studies (magenta) and their corresponding orthologs (light blue) detected in insect cells and/or human cells [5–7]. The figure was generated Microsoft Excel using the SankeyArt GmbH plug-in (https://www.sankeyart.com/excel/).

*Orthoflaviviruses* utilize receptor-mediated endocytosis to gain access to host cells. It is proposed that attachment molecules recruit and/or concentrate virus particles at the surface of the cell, thereby facilitating engagement with a primary receptor and eventual endocytosis. Numerous putative receptors have been identified in mammalian systems including C-type lectins (DC-SIGN, L-SIGN, mosGCTL-1/3/7, mannose receptor), phosphatidylserine receptors (TIM/TAM families, CD300a), integrins receptors (αvβ3, αvβ5), glycosaminoglycans (heparan sulfate, heparan sulfate proteoglycan), laminin receptors (heat shock proteins, 37/67 kDa receptor), and claudin-1/prohibitin-1 [5,6]. These findings suggest that orthoflaviviruses have evolved mechanisms to leverage a broad spectrum of molecules to interact with mammalian cells (Fig 1). It should be noted that many of these putative receptors have been shown to mediate virus attachment to host cells, but not necessarily internalization which is a prerequisite for bona fide receptors. Notably, mosquitoes lack orthologues for many of these molecules.

Similarly, several mosquito receptors have been shown to facilitate viral entry in both mosquito cell lines and *Aedes aegypti*. Key examples include prohibitin which interacts with DENV, CLEC5A, and mosGCTL to mediate JEV, DENV, and ZIKV attachment, and a C-Type lectin in collaboration with a CD45 phosphatase homolog which facilitate WNV infection in mosquitoes [6,7]. Moreover, additional attachment factors (50, 67, and 80 kDa proteins) present in C6/36 cells and the midgut of *Ae. aegypti* influence DENV attachment in a serotype-specific manner [5]. It has recently been reported that a 31 kDa protein serves as a receptor for DENV2 in *Ae. aegypti* mosquitoes [8]. Interestingly, this protein was found to localize intracellularly, and partial knockout or dsRNA suppression of this gene had no effect on infection prevalence. Prevalence was only reduced upon dsRNA suppression in the heterozygous knockout line [8]. These data suggest a role for this 31 kDa protein during infection, but fail to definitively validate it as a bona fide receptor. Moving forward, it will be important that putative receptors, in both vertebrate and invertebrate systems (Fig 1), be validated with overexpression experiments in nonpermissive cells/hosts, knockout experiments, binding assays, and inhibition assays.

**Fig 2:**
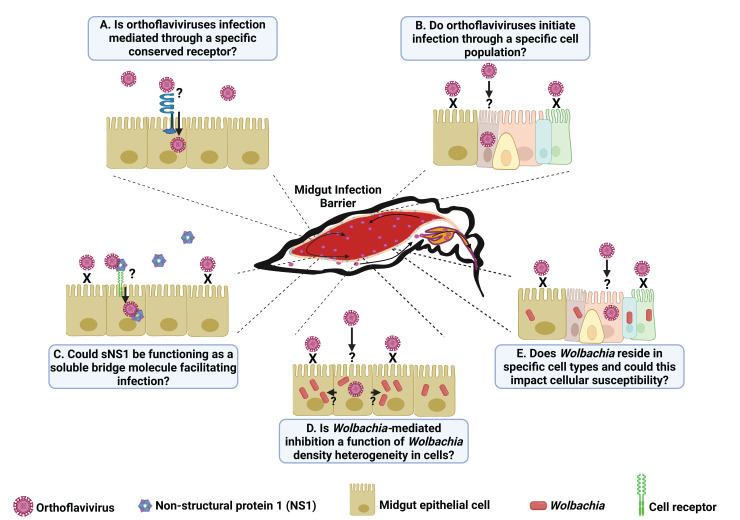
Multifactorial influences on mosquito midgut infection. An illustration depicting arbovirus midgut infection barrier in *Aedes aegypti* mosquitoes. It is known that midgut infection is initiated through only a handful of cells, but the reasons why are unclear. Here, we highlight five poorly defined factors potentially involved in mediating orthoflavivirus infection of mosquitoes. **(A)** Orthoflaviviruses utilize receptor-mediated endocytosis to infect cells; however, a bona fide receptor has yet to be identified, and it is unclear if such a receptor is homogeneously expressed across the midgut epithelium. **(B)** The mosquito midgut contains four primary cell types and numerous subpopulations, and it is unknown whether orthoflavivirus utilize a specific cell type to initiate midgut infection. (C) sNS1 is highly conserved and has been shown to enhance viral infection in mosquitoes, but the mechanism of enhancement is unresolved. Evidence suggests that sNS1 may act as a bridging molecule facilitating interactions between extracellular virus particles and the midgut epithelium. **(D, E)** *Wolbachia-*mediated inhibition of orthoflavivirus infection of mosquitoes is well described, but it is unclear if inhibition occurs at the point of infection, during replication, or during cell-to-cell spread. Further, it is unknown if *Wolbachia* infection of the midgut epithelium is homogenous (D) or variable across cell types (E) and the implications of this on orthoflavivirus infection and inhibition. The image was *Created in BioRender. Brackney,* ***D.*** *(2025)* https://biorender.com/dkr7waj.

*Orthoflaviviruses*, like all arthropod-borne (arbo)viruses, are maintained in a transmission cycle between two highly divergent hosts, namely the invertebrate vector and vertebrate host. Consequently, the utilization of a receptor conserved between insect and mammalian hosts would be evolutionarily advantageous. Emerging evidence suggests that alphaviruses such as Western equine encephalitis virus, Semliki Forest virus, Sindbis virus, and Eastern equine encephalitis virus utilize low-density lipoprotein receptor (LDLR), very low-density lipoprotein receptor, and apolipoprotein E receptor 2 and their invertebrate orthologues as receptors for entry into mammalian and mosquito cells [9,10]. Similarly, members of the *Flaviviridae*, including hepatitis C virus (HCV; *Hepacivirus*), DENV, and JEV, have been shown to utilize lipoprotein receptors, specifically LDLR and scavenger receptor class B type 1, to gain entry into mammalian cells [11,12]. The conservation of lipoprotein receptors from mammals to mosquitoes, coupled with the affinity of *Flaviviridae* and *Togaviridae* for these proteins, raises the possibility that lipoprotein receptors may be important in mediating orthoflavivirus infection of mosquitoes. While viruses can utilize multiple receptors to enter different cell types, it would seem evolutionarily advantageous for viruses infecting disparate hosts like vertebrates and invertebrates to utilize a shared or highly similar molecule as a receptor in both hosts (Fig 2A). Future studies characterizing lipoprotein receptor-orthoflavivirus interactions could reveal new targets for the development of novel vector control interventions.

## Midgut cellular diversity and viral tropism

Single-cell RNA sequencing (scRNAseq) technology has revolutionized our understanding about the cellular composition of the mosquito midgut. The utilization of scRNAseq to profile cellular heterogeneity in mosquito tissues indicates that a noninfectious blood meal induces dramatic changes in midgut cellular composition [2,13]. The midgut is composed of roughly 5,000–10,000 cells which are organized into several epithelial cell types including the undifferentiated progenitors (intestinal stem cells, and the enteroblasts (EB)), absorptive enterocytes (EC), and the secretory enteroendocrine cells (EE) [2,13]. The ECs constitute nearly 70% of the total cell population. Based on scRNAseq data, EC and EB cell populations can be further delineated into distinct subtypes such as EC-like-1, EC-like-2, EC-like-3, dEB-1, and dEB-2, among others [2,13]. The origin of these cell subtypes is unknown. It is hypothesized that changes in the midgut transcriptional profile due to shift in diet from sugar to blood could result in the formation of transient differentiating cells that occur as distinct metabolic states. Investigations of the interaction between midgut epithelial cells and viral pathogens suggest that EC-like-2 cells are the most susceptible to West Nile virus infection, while Zika virus is more commonly associated with EC and EE cells [1,14]. These studies provide an early indication of a possible link between cell types and virus infection (Fig 2B); however, these investigations only focused on established infections (4 and 12 dpi) and not early time points during the initial stages of infection (i.e., 6–8 hpi) [1,14]. This is significant because during an infection, extracellular viruses infect a susceptible cell and then spread cell-to-cell forming foci. These two modes of transmission are fundamentally different and, therefore, insights gained from established infections may not be relevant to the initial infection events. Further, it is known that the acquisition of a blood meal can drastically shift the cellular composition and metabolic state of the midgut meaning cell populations present at the time of infection may not be the same as those present days later after the blood meal has been digested. Examining earlier time points will be critical to determining if orthoflaviviruses preferentially initiate infection of a specific cell type or metabolic state.

## Gut feeling: How NS1 supercharges midgut infections

The envelope glycoprotein of orthoflaviviruses (e.g., DENV, ZIKV, WNV, and YFV) has long been considered the viral factor responsible for host cell attachment and penetration; however, recent work suggests that the multifunctional and highly conserved NS1 may also play an important role in initiating infection [15,16]. NS1 is essential for virus replication and has both intracellular (iNS1) and secreted (sNS1) forms. The intracellular dimeric iNS1 plays a critical role during virus replication by remodeling ER membranes resulting in the formation of viral replication compartments [17]. The secreted form is not critical to viral replication but has been shown to directly interact with components of the innate immune system (complement factors and toll-like receptors) and modulate aspects of the host’s innate immune response [18]. Further, it can directly interact with endothelial cells resulting in increased expression/activation of cathepsin L, heparinase, and sialidase which can cause disruption of the endothelial glycocalyx layer inducing hyperpermeability [19]. It has also been shown that clinically relevant serum levels of sNS1 can enhance orthoflavivirus infection of the mosquito midgut [20,21]. Mechanistically, it was reported that enhancement occurs through sNS1 suppression of ROS production within the midgut epithelial cells. At 18 hours post sNS1 exposure (hpe), it was found that H_2_O_2_ levels were decreased, as well as the expression levels of dual oxidase and NADPH oxidase transcripts in the midguts that were exposed sNS1 [20]. Interestingly, similar results were not observed during the early events of midgut infection (i.e., 4 and 8 hpe). Because virus infection of the midgut occurs within hours of blood meal acquisition, the proposed model implies that virus particles enter cells and begin replicating, but the infection is ultimately cleared upon ROS induction which is subsequently quenched by sNS1 allowing for infection to occur more efficiently. While this study and others have demonstrated that elevated levels of ROS at the point of infection can correlate with decreased infection rates, it is unclear if increased ROS after viral entry has any effect on viral replication [20]. In fact, studies have found that viruses across multiple viral families including the *Flaviviridae* can induce and benefit from an induced ROS state [22]. While decreased levels of ROS were observed at 18 hours postNS1 exposure, it is possible that midgut infection could have occurred at much earlier time points prior to any reductions in ROS [20]. An alternative hypothesis is that sNS1 is acting as a soluble bridge between specific cellular attachment molecules and the virus (Fig 2C), thereby enhancing infection in both mammalian and insect cells [23]. In fact, it has been shown that sNS1 can directly interact with virus particles and that sNS1 can enhance orthoflavivirus attachment to both mammalian and mosquito cells [23]. Further investigation into the mechanisms involved in sNS1-mediated enhancement are warranted.

## Silent gatekeeper: *Wolbachia* inhibition of *Orthoflavivirus* infection of the midgut

*Wolbachia pipientis* is an obligate intracellular bacterium that exists in different strains with varied ability to render transinfected mosquito populations resistant to orthoflavivirus infection. The mechanism(s) of inhibition are not fully resolved, but host immune priming, resource competition, and metabolic alterations are thought to contribute to this phenotype [24]. Further, there are many strains of *Wolbachia* each inducing varying degrees of inhibition. For example, *wMel* strongly inhibits DENV2 infection of the *Ae. aegypti* midgut whereas *wAlbB* has an intermediate inhibitory effect [25]. While numerous studies have demonstrated that *wMel* inhibits infection, this observation has been made at later time points post virus exposure, and it is therefore impossible to discern if inhibition occurs at the point of infection or during cell-to-cell spread [26]. It is known that *Wolbachia* bacteria can stably reside in midgut tissue, but it is unclear if all cells are equally infected at similar densities or if *Wolbachia* reside in specific cell types within the midgut epithelium, and how this affects the inhibitory phenotype. For instance, it has been shown that *Wolbachia*-mediated virus inhibition can occur in a density-dependent manner; however, these studies assessed *Wolbachia* densities at a tissue or organismal level and not the cellular level or were completed in cell culture [27–29]. If *Wolbachia* densities are heterogeneous across midgut epithelial cells this could affect the ability of orthoflaviviruses to establish midgut infections by limiting the number of susceptible cells or by preventing initiated infections from spreading cell to cell (Fig 2D). Alternatively, *Wolbachia spp.* or specific *Wolbachia* strains could preferentially infect specific cell types within the midgut epithelium. If the preferentially infected cell type is important for viral infection it could result in variable infection phenotypes (Fig 2E). Understanding these diverse cellular processes may further explain the complex interactions between *Wolbachia* and mosquito hosts, thereby elucidating how different strains influence host biology, physiology, and vector competence. To address these unknowns, future studies should focus on analyzing the midguts of orthoflavivirus-exposed *Wolbachia*-transinfected mosquitoes across a series of early time points (e.g., 6, 12, 18, and 24 hpi) through advanced signal amplifying immunofluorescence techniques (e.g., hybridization chain reaction) and scRNAseq.

## Concluding remarks

The recent application of high-throughput screens and single-cell sequencing technologies has significantly advanced our understanding of virus-vector interactions; however, the critical viral and/or host factors mediating the initial infection events remain unresolved. Future work should focus on identifying and validating potential receptors, most likely orthologous genes present in both the vertebrate host and invertebrate vector, clarifying the role of specific cellular population/subpopulations during infection, determining the mechanism by which sNS1 enhances midgut infection, and how *Wolbachia* effects the early stages of midgut infection. The results from these studies will be crucial for developing novel control strategies that disrupt viral infection of the mosquito midgut.

## References

[1] Chen T-Y, Raduwan H, Marín-López A, Cui Y, Fikrig E. Zika virus exists in enterocytes and enteroendocrine cells of the *Aedes aegypti* midgut. iScience. 2024;27(7):110353. doi: 10.1016/j.isci.2024.110353 39055935 PMC11269924

[2] Cui Y, Franz AWE. Heterogeneity of midgut cells and their differential responses to blood meal ingestion by the mosquito, *Aedes aegypti*. Insect Biochem Mol Biol. 2020;127:103496. doi: 10.1016/j.ibmb.2020.103496 33188922 PMC7739889

[3] Scholle F, Girard YA, Zhao Q, Higgs S, Mason PW. trans-Packaged West Nile virus-like particles: infectious properties in vitro and in infected mosquito vectors. J Virol. 2004;78(21):11605–14. doi: 10.1128/JVI.78.21.11605-11614.2004 15479801 PMC523254

[4] Smith DR, Adams AP, Kenney JL, Wang E, Weaver SC. Venezuelan equine encephalitis virus in the mosquito vector *Aedes taeniorhynchus*: infection initiated by a small number of susceptible epithelial cells and a population bottleneck. Virology. 2008;372(1):176–86. doi: 10.1016/j.virol.2007.10.011 18023837 PMC2291444

[5] Cruz-Oliveira C, Freire JM, Conceição TM, Higa LM, Castanho MARB, Da Poian AT. Receptors and routes of dengue virus entry into the host cells. FEMS Microbiol Rev. 2015;39(2):155–70. doi: 10.1093/femsre/fuu004 25725010

[6] Liu J, Quan Y, Tong H, Zhu Y, Shi X, Liu Y, et al. Insights into mosquito-borne arbovirus receptors. Cell Insight. 2024;3(6):100196. doi: 10.1016/j.cellin.2024.100196 39391003 PMC11462183

[7] Cheng G, Cox J, Wang P, Krishnan MN, Dai J, Qian F, et al. A C-type lectin collaborates with a CD45 phosphatase homolog to facilitate West Nile virus infection of mosquitoes. Cell. 2010;142(5):714–25. doi: 10.1016/j.cell.2010.07.038 20797779 PMC2954371

[8] Kantor AM, Talyuli OAC, Reid WR, Alvarenga PH, Booker J, Lin J, et al. Identification of a dengue 2 virus envelope protein receptor in *Aedes aegypti* critical for viral midgut infection. Proc Natl Acad Sci U S A. 2024;121(48):e2417750121. doi: 10.1073/pnas.2417750121 39565309 PMC11621822

[9] Clark LE, Clark SA, Lin C, Liu J, Coscia A, Nabel KG, et al. VLDLR and ApoER2 are receptors for multiple alphaviruses. Nature. 2022;602(7897):475–80. doi: 10.1038/s41586-021-04326-0 34929721 PMC8808280

[10] Ma H, Adams LJ, Raju S, Sariol A, Kafai NM, Janova H, et al. The low-density lipoprotein receptor promotes infection of multiple encephalitic alphaviruses. Nat Commun. 2024;15(1):246. doi: 10.1038/s41467-023-44624-x 38172096 PMC10764363

[11] Cosset F-L, Denolly S. Lipoprotein receptors: a little grease for enveloped viruses to open the lock?. J Biol Chem. 2024;300(11):107849. doi: 10.1016/j.jbc.2024.107849 39357828 PMC11550601

[12] Heaton NS, Randall G. Multifaceted roles for lipids in viral infection. Trends Microbiol. 2011;19(7):368–75. doi: 10.1016/j.tim.2011.03.007 21530270 PMC3130080

[13] Vial T, Lopez-Maestre H, Couderc E, Pinaud S, Howick V, Akorli J, et al. Single-cell transcriptional landscapes of *Aedes aegypti* midgut and fat body after a bloodmeal. Cell Genom. 2025;5(8):100924. doi: 10.1016/j.xgen.2025.100924 40570845 PMC12366660

[14] Fitzmeyer EA, Dutt TS, Pinaud S, Graham B, Gallichotte EN, Hill JL, et al. A single-cell atlas of the Culex tarsalis midgut during West Nile virus infection. PLoS Pathog. 2025;21(1):e1012855. doi: 10.1371/journal.ppat.1012855 39869679 PMC11793825

[15] Perera DR, Ranadeva ND, Sirisena K, Wijesinghe KJ. Roles of NS1 protein in flavivirus pathogenesis. ACS Infect Dis. 2024;10(1):20–56. doi: 10.1021/acsinfecdis.3c00566 38110348

[16] Rastogi M, Sharma N, Singh SK. Flavivirus NS1: a multifaceted enigmatic viral protein. Virol J. 2016;13:131. doi: 10.1186/s12985-016-0590-7 27473856 PMC4966872

[17] Shu B, Ooi JSG, Tan AWK, Ng T-S, Dejnirattisai W, Mongkolsapaya J, et al. CryoEM structures of the multimeric secreted NS1, a major factor for dengue hemorrhagic fever. Nat Commun. 2022;13(1):6756. doi: 10.1038/s41467-022-34415-1 36347841 PMC9643530

[18] Modhiran N, Watterson D, Blumenthal A, Baxter AG, Young PR, Stacey KJ. Dengue virus NS1 protein activates immune cells via TLR4 but not TLR2 or TLR6. Immunol Cell Biol. 2017;95(5):491–5. doi: 10.1038/icb.2017.5 28220810

[19] Puerta-Guardo H, Glasner DR, Harris E. Dengue virus NS1 disrupts the endothelial glycocalyx, leading to hyperpermeability. PLoS Pathog. 2016;12(7):e1005738. doi: 10.1371/journal.ppat.1005738 27416066 PMC4944995

[20] Liu J, Liu Y, Nie K, Du S, Qiu J, Pang X, et al. Flavivirus NS1 protein in infected host sera enhances viral acquisition by mosquitoes. Nat Microbiol. 2016;1(9):16087. doi: 10.1038/nmicrobiol.2016.87 27562253 PMC5003325

[21] Liu Y, Liu J, Du S, Shan C, Nie K, Zhang R, et al. Evolutionary enhancement of Zika virus infectivity in *Aedes aegypti* mosquitoes. Nature. 2017;545(7655):482–6. doi: 10.1038/nature22365 28514450 PMC5885636

[22] Zhang Z, Rong L, Li Y-P. Flaviviridae viruses and oxidative stress: implications for viral pathogenesis. Oxid Med Cell Longev. 2019;2019:1409582. doi: 10.1155/2019/1409582 31531178 PMC6720866

[23] Dibiaso White MJ. Structure-function analysis of a flavivirus nonstructural protein. Open Access Dissertations. Purdue University; 2018.

[24] Cook PE, McGraw EA. Wolbachia pipientis: an expanding bag of tricks to explore for disease control. Trends Parasitol. 2010;26(8):373–5. doi: 10.1016/j.pt.2010.05.006 20647151

[25] Johnson RM, Breban MI, Nolan BL, Sodeinde A, Ott IM, Ross PA, et al. Implications of successive blood feeding on Wolbachia-mediated dengue virus inhibition in *Aedes aegypti* mosquitoes. Nat Commun. 2025;16(1):6971. doi: 10.1038/s41467-025-62352-2 40730791 PMC12307751

[26] Novelo M, Audsley MD, McGraw EA. The effects of DENV serotype competition and co-infection on viral kinetics in Wolbachia-infected and uninfected *Aedes aegypti* mosquitoes. Parasit Vectors. 2021;14(1):314. doi: 10.1186/s13071-021-04816-0 34108021 PMC8190863

[27] Bian G, Xu Y, Lu P, Xie Y, Xi Z. The endosymbiotic bacterium Wolbachia induces resistance to dengue virus in *Aedes aegypti*. PLoS Pathog. 2010;6(4):e1000833. doi: 10.1371/journal.ppat.1000833 20368968 PMC2848556

[28] Frentiu FD, Robinson J, Young PR, McGraw EA, O’Neill SL. Wolbachia-mediated resistance to dengue virus infection and death at the cellular level. PLoS One. 2010;5(10):e13398. doi: 10.1371/journal.pone.0013398 20976219 PMC2955527

[29] Lu P, Bian G, Pan X, Xi Z. Wolbachia induces density-dependent inhibition to dengue virus in mosquito cells. PLoS Negl Trop Dis. 2012;6(7):e1754. doi: 10.1371/journal.pntd.0001754 22848774 PMC3404113

